# Phenotypic heterogeneity in persisters: a novel ‘hunker’ theory of persistence

**DOI:** 10.1093/femsre/fuab042

**Published:** 2021-08-06

**Authors:** J Urbaniec, Ye Xu, Y Hu, S Hingley-Wilson, J McFadden

**Affiliations:** Department of Microbial Sciences and University of Surrey, Guildford, Surrey, GU27XH, UK; Department of Microbial Sciences and University of Surrey, Guildford, Surrey, GU27XH, UK; Farnborough Sensonic limited, Farnborough road, GU14 7NA, UK; Department of Microbial Sciences and University of Surrey, Guildford, Surrey, GU27XH, UK; Department of Microbial Sciences and University of Surrey, Guildford, Surrey, GU27XH, UK; Quantum biology doctoral training centre, University of Surrey, Guildford, Surrey, GU27XH, UK

**Keywords:** antibiotic persistence, antibiotic, antimicrobial resistance (AMR), *Escherichia coli*, *Mycobacterium*, microbial heterogeneity

## Abstract

Persistence has been linked to treatment failure since its discovery over 70 years ago and understanding formation, nature and survival of this key antibiotic refractory subpopulation is crucial to enhancing treatment success and combatting the threat of antimicrobial resistance (AMR). The term ‘persistence’ is often used interchangeably with other terms such as tolerance or dormancy. In this review we focus on ‘antibiotic persistence’ which we broadly define as a feature of a subpopulation of bacterial cells that possesses the non-heritable character of surviving exposure to one or more antibiotics; and persisters as cells that possess this characteristic. We discuss novel molecular mechanisms involved in persister cell formation, as well as environmental factors which can contribute to increased antibiotic persistence *in vivo*, highlighting recent developments advanced by single-cell studies. We also aim to provide a comprehensive model of persistence, the ‘hunker’ theory which is grounded in intrinsic heterogeneity of bacterial populations and a myriad of ‘hunkering down’ mechanisms which can contribute to antibiotic survival of the persister subpopulation. Finally, we discuss antibiotic persistence as a ‘stepping-stone’ to AMR and stress the urgent need to develop effective anti-persister treatment regimes to treat this highly clinically relevant bacterial sub-population.

## Teaser

This review discusses recent developments in the field of antibiotic persistence, and aims to provide a novel, comprehensive ‘hunker’ theory of persister cell formation based on intrinsic heterogeneity of bacterial growth and metabolism.

## INTRODUCTION

### Discovery of persistence

The phenomenon of antibiotic persistence was first observed by an American microbiologist Gladys Hobby who observed that a small fraction of *Streptococcus* cells was able to survive penicillin treatment that proved lethal to the rest of the isogenic population (Hobby, Meyer and Chaffee 1942). Shortly after, in 1944, the phenomenon was named ‘bacterial persistence’ by an Irish academic Joseph Bigger who observed that a small minority of cells (about 1 in 10^6^) in cultures of *Staphylococcus aureus*, isolated from patients, could not be eradicated even using high doses of penicillin. Bigger also hypothesised that persistence was induced by environmental stresses, such as pH, and might be caused by slow growth rate of persister cells rather than a heritable resistance (Bigger 1944). It would be another forty years before the phenomenon of persistence was further investigated through molecular techniques, largely due to the difficulty of studying minority subpopulations using traditional microbiological methods. Alongside molecular advances, increasing evidence has emerged implicating antibiotic persistence as a cause of treatment failure (Cohen, Lobritz and Collins 2013) and as a ‘stepping-stone’ for genetic antibiotic resistance (Windels *et al*. 2019; Liu *et al*. 2020). Antibiotic persistence is noted in multiple clinically relevant pathogens from *Mycobacterium tuberculosis* (Manina, Dhar and McKinney 2015; Vilcheze and Jacobs 2019), *Pseudomonas aeruginosa* (Nguyen *et al*. 2011), *Escherichia coli* (Balaban *et al*. 2004) and Methicillin-Resistant *S. aureus* (MRSA) (Kim *et al*. 2018) and is likely to be a cornerstone to disease control and to combatting AMR.

### What is a persister?

In this review, the term ‘persistence’ will refer to a subpopulation of bacterial cells that possess the non-heritable character of surviving prolonged exposure to an antibiotic that kills isogenic sister cells. The key features that distinguish persistence from genetic resistance are that it involves only a subpopulation of isogenic cells that do not carry resistance genes, so the phenomenon is sometimes termed as phenotypic, rather than genetic, resistance. It also tends to not be antibiotic-specific; or, at least, persister cells can be detected that are tolerant to a wide variety of antibiotics, suggesting that the majority of persistence is a general, rather than antibiotic-specific, phenomenon. However, it appears that there is a subclass of persisters that are antibiotic-specific, which we will also describe further in this review.

Persisters are also sometimes described as being *tolerant* to an antibiotic, rather than genetically resistant, with the term ‘tolerance’ indicating that the capability of surviving exposure to antibiotics is reversible and non-heritable. In contrast to persistence, antibiotic tolerance can be a feature of an entire population of cells when, for example, it is exposed to stress, starvation or stationary phase growth, which are conditions that restrict growth of the entire population. In this sense, persisters could also be described as being hetero-tolerant, *i.e*. a subpopulation of antibiotic tolerant cells within a population of mostly sensitive cells (Balaban *et al*. 2019). Persistence is also a term used to refer to infections that persist in the host without causing disease (Centre for Disease Control and Prevention 2019) and without reference to antibiotic persistence. Although it has often been speculated that these ‘infection persisters’ are the same kind of cells as antibiotic persisters, this remains to be established. To avoid confusion of terms, Balaban has recently proposed that persisters, meaning a subpopulation of tolerant cells, should, at least initially, be referred to as ‘antibiotic persisters’ (Balaban *et al*. 2019) to distinguish them from infection persisters. A summary of these terms is shown in Table 1. Another distinct subpopulation of cells that can be observed in bacterial culture are viable but non-culturable cells (VBNCs/ sleeper cells) that remain intact and metabolically active after exposure to an antibiotic but, unlike persisters, do not resume growth after removal of the antibiotic (Dong *et al*. 2020). It has been shown that, if incubated for long enough, some VBNCs of several bacterial species may eventually resume growth (Dong *et al*. 2020) and could thereby represent a persister population with a very long lag phase. This assumption is supported by transcriptomics, where it has been demonstrated in an innovative study that *E. coli* ‘sleepers’ display similar levels of expression of several persistence-related genes to persister cells (Bamford *et al*. 2017). However, for the purposes of this review, we will retain the term persisters to cells that both remain viable and eventually regrow after removal of antibiotic. The link between persister formation and persister subclasses within biofilms is another intriguing avenue of research and one which may require further nomenclature changes in the future.

Note that the above persister definition encompasses cells that survive exposure to antibiotics not only *in vitro*, but also during treatment of infections in humans or animal models. The existence of such cells was established as early as 1956 by McCune and Tompset who used a mouse-infection tuberculosis (TB) model to demonstrate that antibiotic-sensitive *M. tuberculosis* bacilli could be cultured from one third of experimental animals 90 days after successful treatment with isoniazid, para-aminosalicylic acid (PAS) and streptomycin (as determined by culture, microscopy and sub-inoculation) (McCune, Tompset and Mcdermott 1956). A shortened revival time was detected in immuno-compromised mice, suggestive of immune-regulation of persistence (McCune *et al*. 1966). Pathogens might survive in a treated host due to lack of penetration of the antibiotic into the persister bacilli or in tissue sites (Ray *et al*. 2015), cell types (Greenwood *et al*. 2019) or through an inadequate host immune response, as in *in utero* infections of bovine viral diarrhoea virus (Khodakaram-Tafti and Farjanikish 2017). However, these explanations are unlikely to account for the persistence of the infection in multiple body sites after prolonged treatment, as in the above mouse model. It seems more likely, and thereby it is generally assumed, that the subpopulation of cells that survive prolonged antibiotic treatment in patients or animal models are indeed the same kind of persister cells that survive prolonged antibiotic exposure *in vitro*. This is an important assumption as it justifies the relevance of *in vitro* persistence models to *in vivo* systems and clinical disease, however to our knowledge it remains unproven.

The McCune and Tompset experiments also revealed another important aspect of persistence: it is not the same for all antibiotics. Although persister cells survived 12 weeks of treatment with isoniazid, PAS and streptomycin in the mouse model, no viable bacteria could be recovered from animals treated for the same period with just pyrazinamide and isoniazid. This suggests that pyrazinamide may be more effective at killing TB persisters, however the exact mechanism through which this occurs is currently unknown. The observation that antibiotics differ in their relative efficacy against persisters vs the bulk population of cells demonstrates that persisters are physiologically distinct from the bulk population. This provides the rationale for the long-term aim of identifying novel compounds that target these physiologically distinct antibiotic-refractory cells in order to shorten treatment regimes.

### What are the evolutionary origins of antibiotic persistence?

Although persistence is non-heritable, the frequency of persister cells in a population is a heritable trait, as has been shown by several studies that demonstrate gene mutations that either increase or decrease that frequency (Moyed and Bertrand 1983; Wolfson *et al*. 1990; Hingley-Wilson *et al*. 2020). Being heritable, the frequency of persistence in the population would be visible to natural selection and likely evolved as a ‘bet hedging’ strategy. ‘Bet hedging’ is an evolutionary survival strategy first described in 1974 (Slatkin 1974). It proposes that in uncertain changing environments it is evolutionarily favourable to introduce phenotypic heterogeneity into an isogenic population to be better prepared for unpredictable environmental perturbations, such as encountering antibiotics (Slatkin 1974). The theory is supported by mathematical models in which populations generating higher persister cell levels have an increased fitness when repeatedly exposed to bactericidal antibiotics (Johnson and Levin. 2013). It also seems to be supported by recent evidence that laboratory exposure of *E. coli* to repeated dual-antibiotic regimes results in significant enrichment of the persister fraction, through convergent mutations in proteins involved in tRNA synthetases, as well as methionine synthetase metG (Khare and Tavazoie 2020). An alternative perspective is that persistence is an inevitable consequence of ‘errors’ during cell cycle and division that introduce phenotypic heterogeneity, a hypothesis sometimes known under the acronym ‘PASH’ (persistence as stuff that happens) (Levin, Concepcion-Acevedo and Udekwu 2014). It could be that different persister classes as described below have different evolutionary origins.

### Can antibiotic persistence facilitate the emergence of genetic antibiotic resistance?

It has recently been hypothesized that this already clinically relevant subpopulation may be a reservoir for genetic antibiotic resistance. For example, prolonged exposure to DNA-damaging fluoroquinolones and hydroxyl radicals produced as a result of exposure to β-lactams, fluoroquinolones and aminoglycosides (Kochanski *et al*. 2007) is likely to lead to a degree of DNA damage, and as a result activation of the SOS response. The SOS response activation in turn causes induction of expression of error-prone DNA polymerases such as DNA polymerase IV & V, which increases rates of adaptive mutation (McKenzie *et al*. 2000), provided that the cell survives the antibiotic exposure (i.e. is a persister). Indeed, there is an increasing array of evidence suggesting that persistence (& whole population antibiotic tolerance) could accelerate genetic resistance development (Vogwill *et al*. 2016; Mok and Brynildsen 2018; Liu *et al*. 2020). For example, there is *in vitro* evidence that persistence can be a contributing factor to the emergence of resistant strains of *M. tuberculosis*, possibly as a result of increased levels of mutagenesis-inducing hydroxyl radicals found in *M. tuberculosis* persisters (Sebastian *et al*. 2017).

Interestingly, persistence to one antibiotic could not only facilitate the emergence of genetic resistance to that antibiotic, but other antibiotics as well. For instance, rifampicin (RIF)-persisters in *M. tuberculosis* which carried elevated levels of hydroxyl radicals were found to carry *de novo* acquired mutations not only associated with RIF-resistance mutations (*rpoB*), but also moxifloxacin-resistant mutations (*gyrA*) (Sebastian *et al*. 2017). Increased mutagenesis rates were also identified in moxifloxacin persisters of *Mycobacterium. smegmatis*, which developed moxifloxacin resistance, as well as resistance to ethambutol and isoniazid (Swaminath *et al*. 2020). A similar mechanism has also been identified in *S. aureus* where exposure to ciprofloxacin resulted in the induction of error prone DNA polymerases that increased the number of resistant mutants 30-fold when compared to a SOS response or error-prone polymerase deficient knock-out control (Cirz *et al*. 2007).

Finally, analysis of clinical isolates of MRSA carried out by Liu and colleagues (Liu *et al*. 2020) has also demonstrated that certain combination therapies which are effective in preventing emergence of genetic AMR through a mechanism called ‘suppressive action’ fail to do so if tolerance to the primary (non ‘suppressive’) drug is established prior or during the course of treatment. Suppressive action is a mechanism by which a combination of two drugs is less effective than the single drug alone, for example in the case of treatment with any β-lactam antibiotic (which most effectively targets dividing cells) and chloramphenicol (which slows down growth) (Jawetz, Gunnison and Speck 1951). This paradoxically decreases the survival rate of chloramphenicol resistant mutants and thereby decreases their frequency in the population. Pre-establishment of tolerance to the non-suppressive antibiotic negates its anti-AMR effect, providing *in-vivo* evidence of a direct link between tolerance (& persistence), resistance and clinical treatment failure (Liu *et al*. 2020). This study suggests that targeting persisters and inhibiting the persistence state could thereby inhibit the development of genetic AMR.

### How is antibiotic persistence quantified?

**Figure 1.**
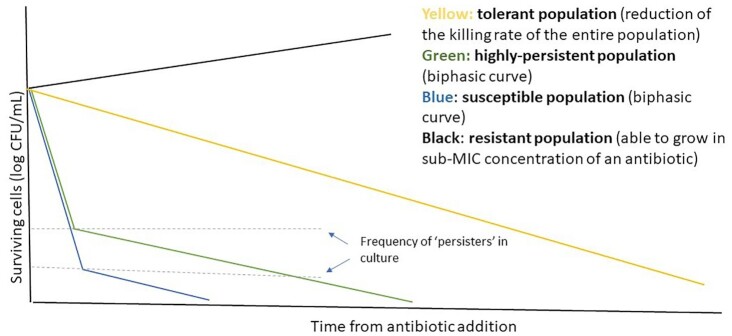
The highly heterogenous persister cell can be observed at batch level. Biphasic killing at batch level: an example time-kill curve shape of resistant, susceptible, high-persister and tolerant cultures. The resistant population is able to grow in sub-inhibitory concentrations of an antibiotic, whereas tolerant & persister cells are eventually killed by the antibiotic, but at a much slower rate. The higher number of persister cells observed in high-persistent strains ensures that the number of cells surviving the initial ‘rapid-killing’ phase of antibiotic exposure is greater than in the susceptible parental strain.

The “gold standard” experimental system used to demonstrate the presence of persisters and estimate their abundance is the antibiotic time-kill experiment, which generates the classic biphasic kill curve from the average kill (Fig. 1). Typically, an exponentially-growing population of isogenic bacteria is exposed to antibiotic and samples are taken at regular time points for determination of colony forming units (cfu, on a log scale) as a function of time. Under conditions where persistence is detectable, the kill rate of is made up of two components: a fast initial kill characteristic of the susceptible population and a slower kill characteristic of the persister subpopulation. If the slow killing phase is extrapolated to zero time then where it crosses the y axis provides an estimate of the persister fraction in the initial population (Balaban *et al*. 2019). Figure 1 shows an ‘ideal’ difference in kill curve shapes between susceptible, highly-persistent, genetically-resistant and tolerant populations. For instance, a typical time-kill curve of *M. tuberculosis* using front-line antibiotics such as isoniazid follows a biphasic curve, as highlighted in this comprehensive review detailing TB persisters (Vilcheze and Jacobs 2019).

It should, however, be emphasized that kill curves are extraordinarily sensitive to the precise conditions of the experiment and particularly the growth state of the initial population, which can lead to well-known reproducibility issues. This experimental and stochastic variation in each experiment can obscure data from the relatively minute persister subpopulation. Different batches of antibiotic or media, culture age, variation in storage or growth conditions and the presence of resident phages can also influence the shape of kill curves (Harms *et al*. 2017). Culture conditions can also profoundly influence phenotypic heterogeneity (Smith *et al*. 2018) which, as we will later discuss, plays a key role in persistence. It also appears that selection and outgrowth may also affect reproducibility where growth regulation is affected (Urbaniec, unpublished results).

**Figure 2.**
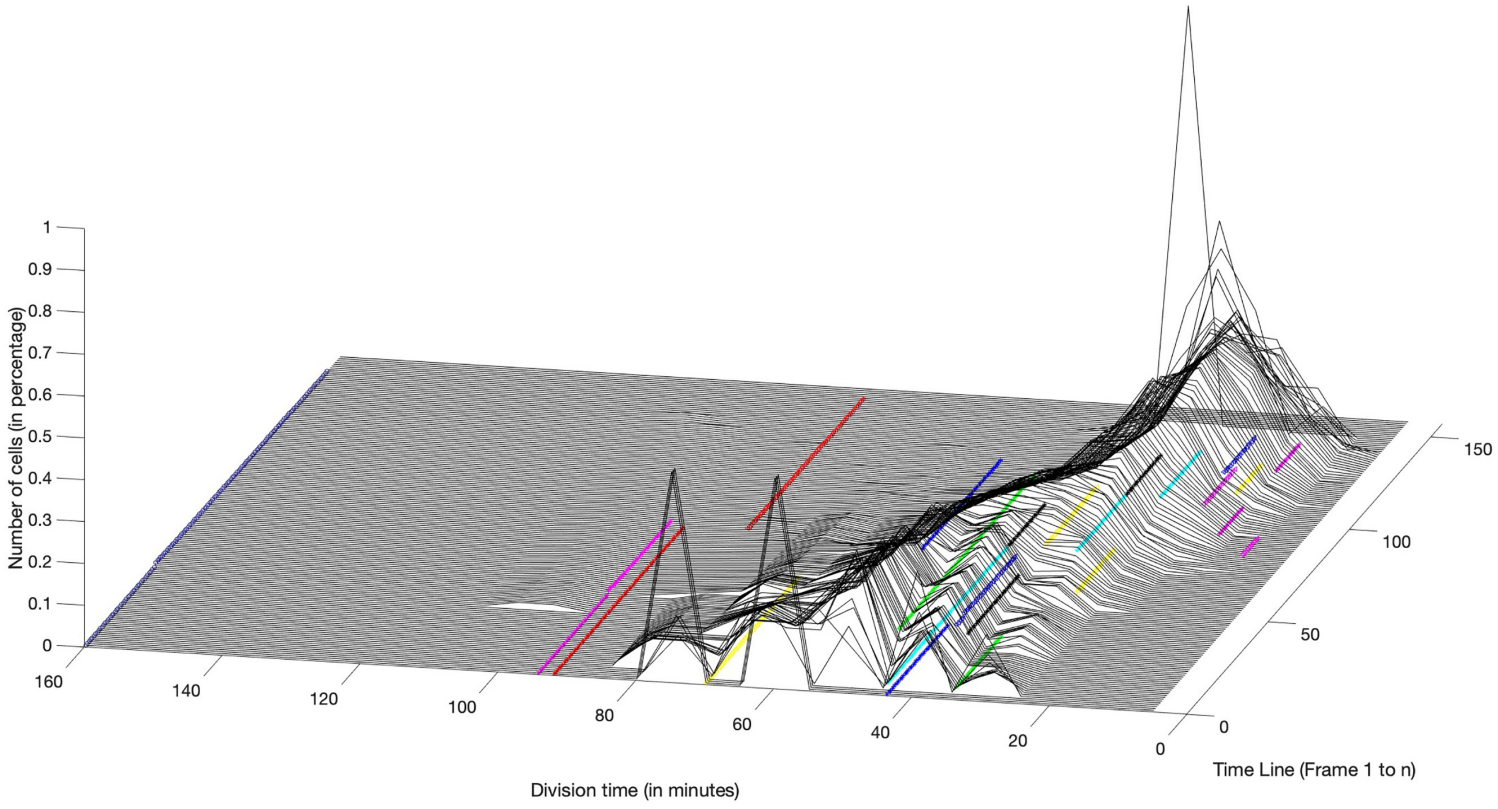
The growth rate of E. coli and the persister's family is highly heterogenous. Single cell microfluidics study showing division time in minutes throughout experimental frames (1 frame = 69 seconds) of the whole population of *E. coli* HipQ cells prior to antibiotic administration (number of cells = 157). Different colours represent different persister ‘families’ (cells from the same lineage). The time point of 160 minutes represents the final time point and also includes non-growing cells (i.e. the blue line represents a non-growing persister cell).

Since, as illustrated by the biphasic kill curve (Fig. 1), persistence is a property of only a minority of cells, traditional microbiological, cell biological or molecular biological analysis techniques that examine bulk populations are unsuitable for studying the phenomenon. Two approaches have been most productively applied to study persistence: microbial genetics and single cell microscopy studies. Indeed, single cell studies from our laboratory show just how heterogenous both microbial populations and persisters family are (Fig. 2). It should be observed in this figure that persisters themselves are only noted on here if they are non-growing (blue line on left hand side) as division time is not measured in these studies following antibiotic administration.

The majority of antibiotic persistence studies have been performed on the microbial workhorse, *E. coli*, which will be discussed first, particularly with reference to the canonical hipA7 system from which much of the theory of persistence has been derived. After examining both genetic and single cell studies of this system we will go on to outline a general theory of persistence and then examine how well the theory accounts for the evidence from a variety of systems.

## ANTIBIOTIC PERSISTENCE IN *ESCHERICHIA COLI*

### Genetic studies to identify *E. coli* hip mutants

The first significant advance in the study of persisters was the application of mutagenesis and microbial genetic studies in the 1980’s to identify ‘high-persister’ or hip mutant strains of *E. coli* K12 strain, including the intensively-studied hipA7 strain (Moyed and Bertrand 1983). In this study, an *E. coli* K12 strain was exposed to untargeted chemical mutagenesis in rich media and then subjected to three rounds of enrichment for mutants that survived repeated exposure to 100 µg of ampicillin, the third exposure being for 20 h. Hip mutants were identified as strains that showed a higher survival after exposure to ampicillin, compared to the wild-type. Mutants that were genetically resistant or unable to grow on minimal media were discarded. It should be noted that mutants with substantially reduced growth of the whole population were also discarded. Note that techniques to examine growth of sub-populations were not yet available. Nonetheless, all the hip mutants identified showed some degree of growth impairment in rich media (Lysogeny broth commonly known as LB), providing a link between mutations increasing persistence and slow growth (Moyed and Bertrand 1983). However, two mutants, HM7 and HM9, grew as rapidly or faster than the parental strain in minimal media yet continued to display 1000-fold higher frequencies of persisters than the wild-type in this media, proving that slow growth of the bulk population is not a requirement of high levels of persistence. The researchers performed batch time-kill assays for wild type and both the hipA7 and hipA9 mutants. Examination of the curves obtained suggested a population of around 0.1% persisters in the wild-type and about ten times this in both mutants. The researchers went on to utilize classical microbial genetic approaches to map the genetic loci for both HM7 and HM9 (Moyed and Bertrand 1983) to the closely-linked loci *hipA7* and *hipA9*.

Later studies (Korch, Henderson and Hill 2003) established that the *hipA7* gene encoded the toxin component of one of several of toxin-antitoxin systems present in the *E. coli* genome. To our knowledge the *hipA9* gene remains uncharacterised. Toxin-antitoxin modules are widely distributed in bacteria and consist of genes encoding a stable toxin and an unstable antitoxin (Ezaki *et al*. 1991). The wild-type *hipA* gene encodes the toxin, hipA, which is neutralized by its antitoxin, hipB, encoded by the adjacent *hipB* gene. If not neutralized, hipA acts as a serine-threonine kinase that inhibits protein synthesis by phosphorylating (and therefore inactivating) glutamyl-tRNA synthetase (GltX), responsible for attachment of glutamine to tRNA. The protein inhibition results in accumulation of uncharged tRNA which indirectly increases levels of (p)ppGpp (guanosine pentaphosphate) in the cell. (p)ppGpp is an alarmone (i.e. a signaling molecule produced in unfavourable conditions) and component of the stringent response in response to amino acid starvation. Downstream effects of its increase include inhibition of RNA synthesis and initiation of growth arrest to induce a state of “dormancy”, understood as a reversible state of little-to-no growth and lower metabolic activity (Semanjski *et al*. 2018). ppGpp levels are themselves regulated by proteins collectively referred to as RSH (RelA SpoT homologs) that are upregulated by various stress-related molecules as the aforementioned uncharged tRNA or cAMP. The hipAB system thereby connects with a wide range of cellular physiological systems (Atkinson, Tenson and Hauryliuk ).

Evidence for hipA toxin-induced growth arrest has been obtained from several systems. For example, Korch and colleagues discovered that over-expression of hipA from a plasmid induces a VBNC-like state in approximately 95% of the population that continued for up to 72hrs with continuous hipA expression. A fraction of cells, from 1.8% to 20% resumed growth following the 72hrs of continuous induction decreasing with increasing induction time. Additionally, in cultures overexpressing HipA a significant (10 000-fold) increase in the number of persisters to ampicillin was observed (Korch and Hill 2006). It was hypothesized that the much lower fraction of persisters in the wild-type population is accounted for by lower levels of hipA expression, similar to that of the HipB antitoxin.

**Figure 3.**
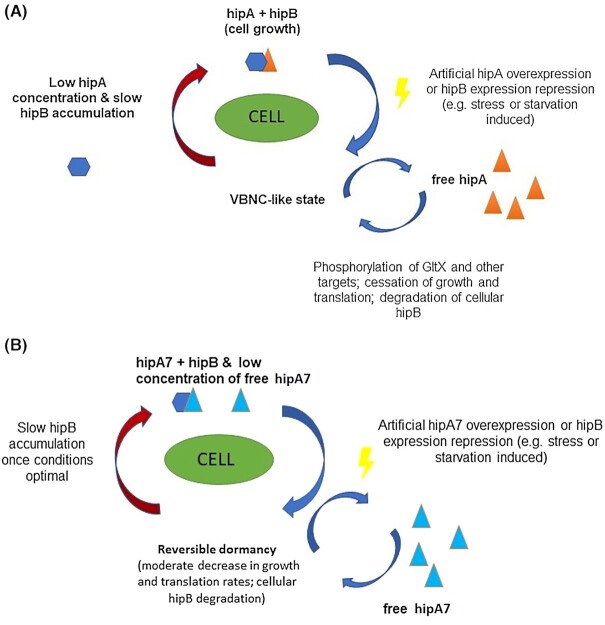
Schematic representation of A. hipA and B. hipA7-induced changes in cellular metabolism. **(A)** hipA phosphorylates *GltX*, as well as several other proteins involved in growth & translation regulation, inducing a HipA/HipB ratio-dependent VBNC-like state in cells containing unbound hipA. Growth can resume only after hipB binds to and inactivates free hipA. **(B)** The mutant hipA7 protein (lower panel) can exist in the unbound form at higher levels in the cell without inducing a VBNC-like state as it only phosphorylates GltX and with lower efficiency than the wild-type protein. High levels of free hipA7 in the cell induce reversible “dormancy” which is proposed to be responsible for the high-persistence phenotype.

The hipA7 high-persistence phenotype is conferred by point mutations at two separate sites in the *hipA* gene (hipA7 allele), both of which are necessary for the hip phenotype (Moyed and Bertrand 1983). How these mutations lead to high levels of persistence is not fully clear (Fig. 3A and B). One appears to render the hipA protein non-toxic since its overexpression only moderately inhibits growth & translation in comparison to wild-type hipA, while the other is required for the observed ‘high-persistence’ phenotype (Korch, Henderson and Hill 2003). The non-toxicity is thought be a consequence of a reduced inability of the hipA7 protein to phosphorylate targets such as GltX and several other proteins involved in regulation of transcription (Semanjski *et al*. 2018). It also has a weaker binding affinity to its cognate antitoxin. However, and rather paradoxically over-expression of the hipA7 allele causes a 12-fold higher phosphorylation of GltX when compared to the wild-type toxin (Semanjski *et al*. 2018). A model has been proposed to account for these apparently contradictory findings, in which chromosomally expressed HipA7 exists more abundantly in the unbound form when compared to hipA, allowing phosphorylation of more GltX. This results in the slower growth rate of hipA7 expressing strain (however this phenotype is lost whenever hipA is artificially overexpressed). Additionally, hipA also phosphorylates other targets which are proposed to contribute to its toxicity (Semanjski *et al*. 2018) and ability to induce the VBNC state (Korch, Henderson and Hill 2003). Interestingly, naturally occurring hipA7 variants has been identified in urinary tract infections suggesting that the locus may also induce persistence *in vivo* (Schumacher *et al*. 2015).

It is important to note that although both hipA and hipA7 alleles have primarily been investigated in relation to persistence to ampicillin, there is a evidence that these toxins contribute to multidrug tolerance. For instance, recently identified *E. coli* clinical isolate carrying the HipA7 allele on the bacterial chromosome has been shown to display up to ∼100-fold higher persistence to fluoroquinolone ciprofloxacin when compared to its parental strain (Schumacher *et al*. 2015). A similar effect has also been observed previously in lab-adapted *E. coli* MG1655 cells induced to overexpress the wild-type hipA from a plasmid (Germain *et al*. 2013). This observed ‘high-persister’ phenotype to fluoroquinolones is presumably a result of toxin-induced extended lag phase of hipA/hipA7 persisters, discussed further in section three of this review.

### Single cell studies of the hipA7 mutant

Although characterization of hip mutants provided insights into factors that might increase or decrease the frequency of persistence, the nature of the persister cells remained a matter of conjecture until pioneering studies performed by Balaban and colleagues who developed microfluidic systems to perform live cell imaging of bacterial populations before, during and after antibiotic exposure. The focus of much of their studies was the hipA7 mutant as its characteristic high frequency of persister cells (1000 x more than wild-type frequencies in their studies) made the phenomenon much more experimentally tractable. The character of persistence was found to be associated with cells that, prior to antibiotic exposure, were either slow-growing or non-growing, prompting the hypothesis that persistence is due to pre-existing phenotypic heterogeneity associated with slow growth rate in the starting population (Balaban *et al*. 2004). The established association between HipA7 and toxin-antitoxin systems prompted the proposal that stochastically variable levels of expression of the hipA toxin and consequent low or zero growth in a sub-fraction of cells might be a source of the pre-existing variation (Korch, Henderson and Hill 2003; Keren *et al*. 2004) and origin of persistence. Thereafter, many other high-persistent mutants in numerous bacterial strains and species have been described (Torrey *et al*. 2016; Wilmaerts *et al*. 2018). Additionally, low persister mutants that have a lower frequency of persisters than wild-type cells have been identified, eg *E. coli ybaL* (Hingley-Wilson *et al*. 2020). Both types of mutants identify genes that influence the frequency of persisters in bacterial populations and thereby provide clues as to the mechanisms of persistence, or, at least, mechanisms involved in the generation of persisters.

**Figure 4.**
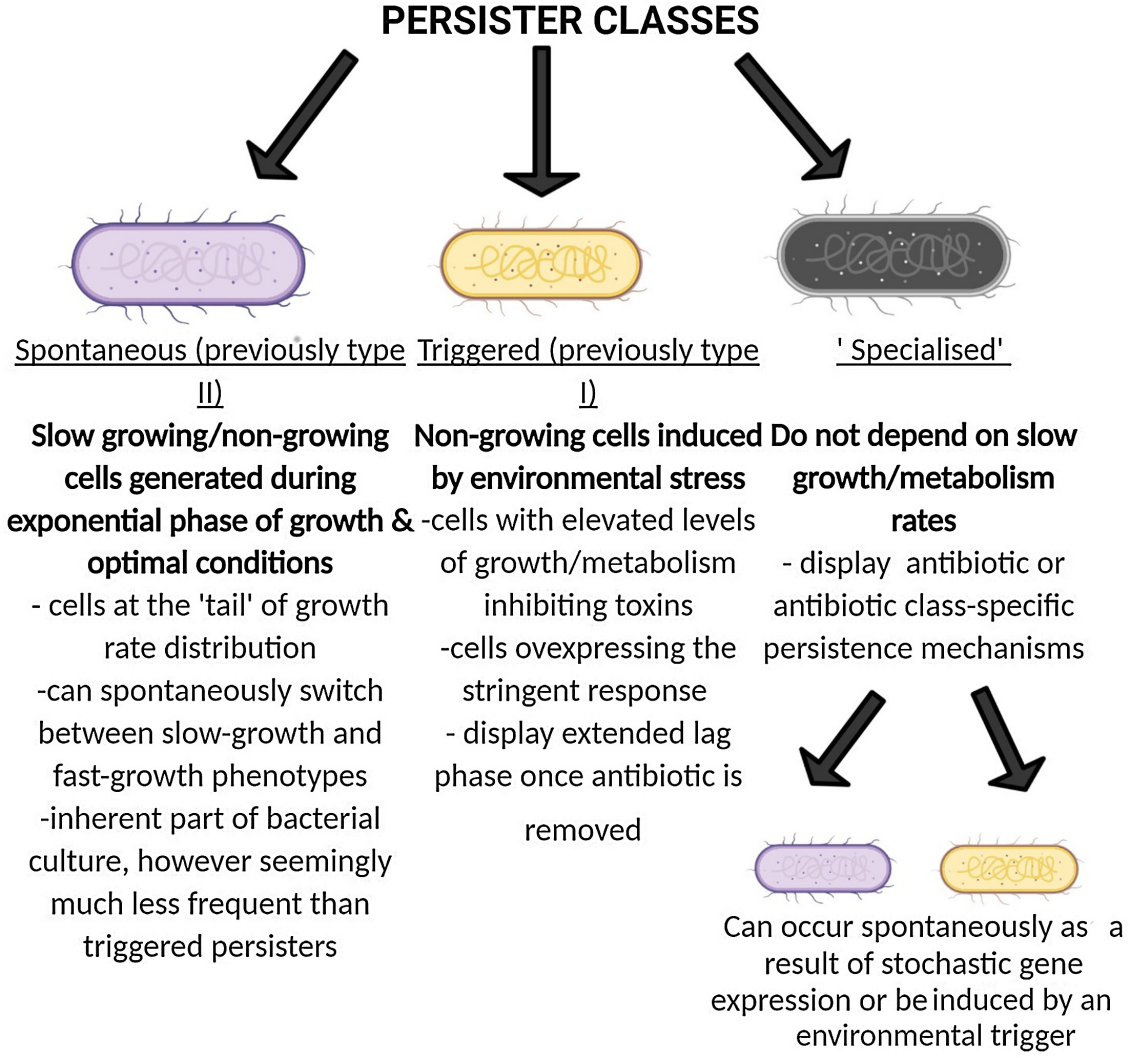
Proposed addition of specialised persisters to classification of persister cells (Balaban *et al*. 2019) (figure created with Biorender.com). Both spontaneous and triggered persisters are slow/non-growing prior to antibiotic addition and often display a general, rather than antibiotic-specific, persistence phenotype. It is, however, important to note that not all slow/non-growing cells are persisters. In contrast, ‘specialised’ persisters do not depend on slow growth/metabolic rates to survive antibiotic exposure, but instead display antibiotic-specific persistence mechanisms. They can occur spontaneously, for example, in *Mycobacteria*, through stochastically low levels of expression of the enzyme catalase-peroxidase that activates isoniazid (Wakamoto *et al*. 2013) or be induced by a stress-signal, for example ciprofloxacin persisters which are induced by exposure of *E. coli* to this antibiotic (Dorr *et al*. 2009; Goormaghtigh and van Melderen 2019).

Moreover, Balaban observed two distinct types of persister cells: triggered (previously type I) persisters which were non-growing cells in stationary phase that displayed extended lag phase upon inoculation into fresh media (presumably caused by nutrient starvation) and stochastic (previously type II) persisters which were slow-growing or non-growing cells generated during exponential phase of growth seemingly without an environmental trigger (Balaban *et al*. 2004; Balaban *et al*. 2019). Persister sub-populations, detected with dual-reporter phages in combination with single cell imaging, were also noted in *M. tuberculosis* following INH treatment as (Jain *et al*. 2016). Interestingly, a potential third class of persisters (referred to here as ‘specialised persisters’) which are not slow-growing prior to antibiotic exposure and often display antibiotic specific persistence mechanisms has also been observed (Wakamoto *et al*. 2013; Goormathigh and Van Melderen 2019), as shown in Fig. 4.

## MOLECULAR MECHANISMS INVOLVED IN PERSISTER CELL FORMATION

### Toxin-antitoxin systems and induction of the stringent response are only one of the many pathways leading to persistence

Inspired by the discovery of the hipA system multiple other TA systems have been linked to the establishment of persistence. HokB toxin expression in *E. coli* is upregulated by the Obg GTPase which is involved in stress response as a regulator of cellular energy levels and whose overexpression results in pore formation in the membrane and ATP leakage from the cell (Wilmaerts *et al*. 2018). TisB toxin, also present in *E. coli*, is involved in a multi-drug tolerance phenotype under the regulation of the SOS response pathway (Dorr, Vulic and Lewis 2010). Another well researched toxin-antitoxin module, MazF-MazE, is induced under stressed conditions and disrupts protein synthesis in *M. tuberculosis* leading to degradation of selected mRNA targets and induction of the stress response operons resulting in growth arrest (Tiwari *et al*. 2015).

However, a fair amount of uncertainty surrounds the effort of developing a unified persistence model based on toxin accumulation, with conflicting results often reported by different research groups. One notable example is a model which aimed to provide a ‘blanket’ pathway on how persistence is established in the model organism *E. coli*. This neat model proposed that increasing levels of ppGpp as a response to external stress causes accumulation of polyphosphate, activation of Lon protease and Lon-mediated accumulation of toxin components of TA systems which in this model acted as effector molecules inducing persistence (Gerdes and Maissoneuve 2012). Although discovery of a single ‘persistence switch’ (in this case Lon-mediated toxin accumulation) would certainly allow for a more systematic approach to persistence research, the ‘real-life’ scenario is likely more complex. The model proposed by Gerdes and colleagues was based on the observations of a decreasing number of persisters following deletions of 10 subsequent TA modules. Unfortunately, some of the data on which this model was based may have been confounded by the presence of resident prophages whose lytic cycle can be induced by an antibiotic (in this case ciprofloxacin) resulting in cell lysis and reduced number of surviving cells, irrespective of persistence mechanisms (Harms *et al*. 2017). This led to the re-evaluation of the role of TA modules in persistence with some groups reporting a link between TA modules and persistence irrespective of ppGpp levels in *E. coli* (Chowdhury, Kwan and Wood 2016) & *M. smegmatis* (Bhaskar *et al*. 2018) and others reporting no link between persistence levels and TA module deletion *in E. coli* (Goormaghtigh *et al*. 2018). The role of TA modules in persistence thereby appears to be more complex than initially reported.

Following Moyed and Bertrand's landmark publication, a significant amount of research has focussed on the (p)ppGpp alarmone system. (p)ppGpp levels are upregulated as a result of various environmental stressors such as nutrient starvation, acid stress or heat shock (Abranches *et al*. 2009), which could act as a trigger in the formation of persister cells, at least in *E. coli* where a link has been established between elevated ppGpp levels in persister cells and nutrient starvation or oxidative stress (Radzikowski *et al*. 2016; Brown 2019). Experiments *on P. aeruginosa*, both *in vitro* and in a mouse infection model, have implicated RelA/SpoT, which synthesize and degrade (p)ppGpp, respectively. A mutant lacking the genes (and therefore incapable of initiation of (p)ppGpp-mediated stringent response) produced 100-fold fewer persisters compared to the wild-type strain (Nguyen *et al*. 2011). The frequency of persisters to multiple antibiotics was also altered in deletion mutants of transcription factors *sigE* or *sigB* (Pisu *et al*. 2017). SigE regulates the transcription of *rel_Mtb_*, which in turn regulates (p)ppGpp (Avarbock *et al*. 2005). Interestingly, deletion of *rel_Mtb_* resulted in reduced (p)ppGpp synthesis and decreased infection persistence of *M. tuberculosis* in a murine model (Dahl *et al*. 2003).

**Figure 5.**
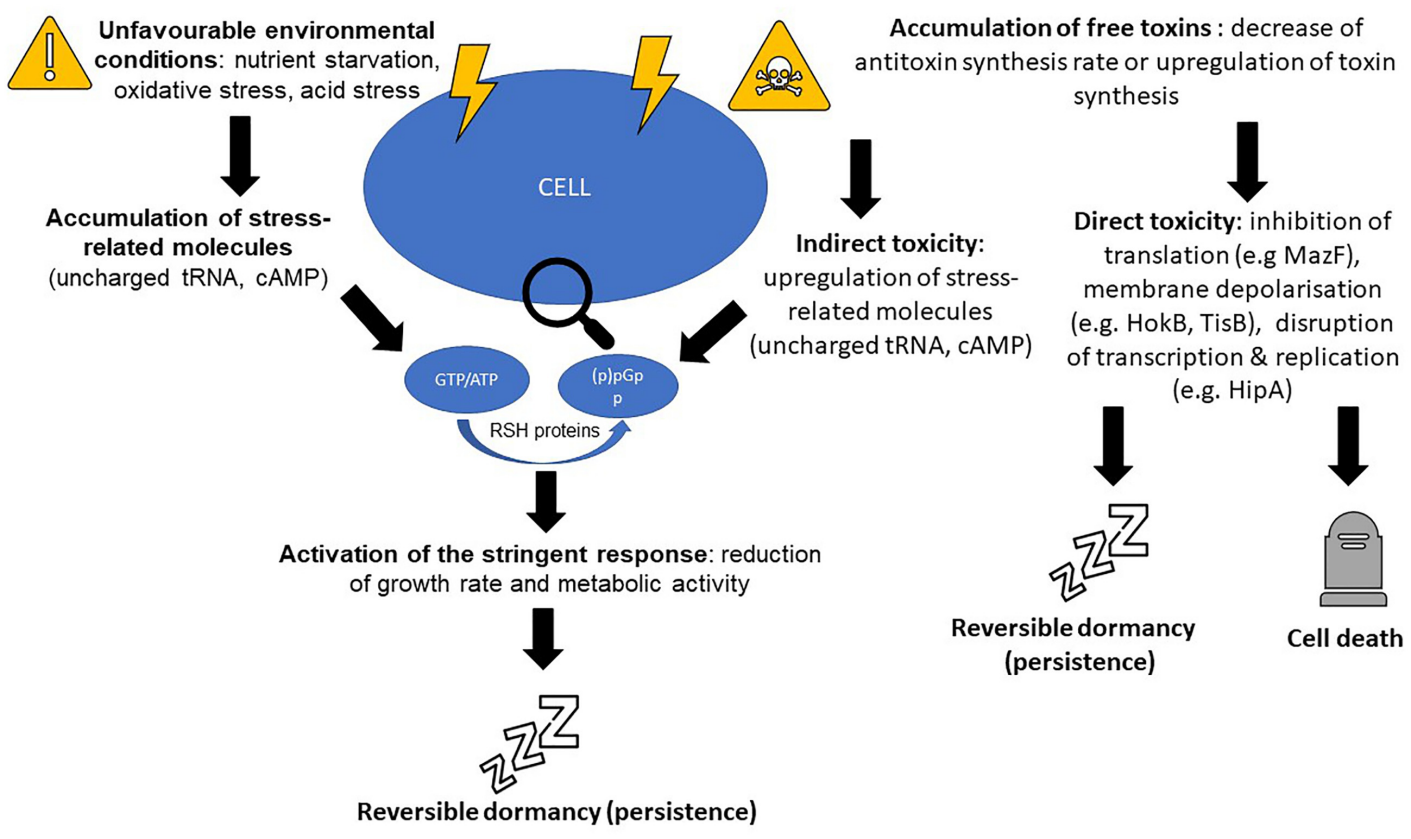
Simplified network displaying the role of the TA systems and the stringent response in establishment of persistence. Cells sense changes in the external environment through receptors such as two-components systems (histidine kinases), thermosensor molecules, pH sensing molecules or GltX inhibition through accumulation of free radicals or phosphorylation by HipA/HipA7. Downstream signaling causes accumulation of stress-related molecules in the cells such as cAMP, uncharged tRNA, heat shock proteins etc. This is sensed by RelA/SpoT proteins which in turn convert ATP/GTP into the (p)ppGpp alarmone. (p)ppGpp affects cellular metabolism directly (by interacting with RNA polymerase) and indirectly (by depleting cellular GTP levels) and has been linked to establishment of the persister state. Toxin components can also directly inhibit crucial metabolic processes such as protein synthesis or replication or affect cellular membrane potential. Depending on the free/bound toxin ratio, this can either result in cell death or temporary growth arrest (persistence).

Taken altogether these findings point towards the hypothesis that persistence is a complex phenomenon potentially involving multiple mechanisms (Fig. 5), both passive (such as growth arrest as a result of toxin accumulation) and active (such as response to oxidative stress). This makes the effort of establishing a comprehensive persistence model a challenging task.

### Specialised persisters

It seems likely that the phenomenon and mechanisms of persistence are much more varied than toxin-antitoxin systems & ppGpp-induced growth arrest. For example, ‘specialised’ persister cells are generated during exponential growth, but are not associated with slow individual cell growth, and are specific to a particular antibiotic (Wakamoto *et al*. 2013; Goormagthigh and van Melderen 2019) and Fig. 4. The antibiotic isoniazid is an inactive prodrug, which becomes activated inside the mycobacterial cells through cleavage by the bacterial enzyme KatG (catalase-peroxidase). Single-cell level observations have shown that, during exposure to isoniazid, the growth/ division rate of a cell does not correlate with persistence. Instead, slowly growing cells were as likely to die as fast-growing cells. Instead, intrinsic noise in gene expression leads to fluctuations in the level of transcription and translation of all cellular genes resulting in cell-to-cell variation of individual gene expression levels (Raj and van Oudenaarden 2008). In mycobacteria, intrinsic noise in *katG* gene expression gave rise to a pulsing of intracellular enzyme activity that appeared to be associated with persistence specific to isoniazid. Even a small increase in *katG* expression was shown to lead to a huge decrease in the number of persister cells and persister cells tended to express less ‘pulsing’ of KatG levels than non-persister cells (Wakamoto *et al*. 2013). Similarly, a single-cell imaging study carried out on *E. coli* has demonstrated that survival to fluoroquinolones in actively growing cells, prior to exposure to the antibiotic, requires appropriate timing of the SOS system induction during the recovery & repair phase and hence, prior to growth resumption. This study demonstrated that fluoroquinolone persisters are highly heterogenous and are not always slow-growing or SOS-induced prior to antibiotic addition as discussed below (Goormaghtigh and Van Melderen 2019).

### Persistence as a result of extended lag phase

Duration of the lag phase can be extended by elevated levels of growth-inhibiting toxin components of the TA systems (such as MazF or hipA) and was found to be an important mechanism for establishment of persistence to fluoroquinolones in *E. coli*. Fluoroquinolones damage the DNA of both actively growing and non-growing cells and therefore, through extension of their lag phase *E. coli* persister cells were able to repair incurred DNA damage prior to growth resumption. This made them much more likely to survive exposure to DNA-damaging antibiotics, such as ciprofloxacin (Mok and Brynildsen 2018). A similar effect was observed when *E. coli* cells were exposed to sub-MIC concentrations of ofloxacin, which significantly (1200-fold) increased the number of persisters upon subsequent exposure to a high concentration of this antibiotic, when compared to cultures only exposed to high concentrations of ciprofloxacin. A proposed model behind this involves ‘priming’ of the SOS response through low-levels of DNA damage, resulting in the overexpression of the TisB toxin and subsequent toxin-induced growth arrest (Dorr, Lewis and Vulic 2009). Both MazF and TisB persisters also displayed a multidrug tolerance phenotype and were tolerant to ampicillin, presumably as a result of their arrested growth state (Dorr, Lewis and Vulic 2009; Mok and Brynildsen 2015). However, there is also supporting evidence that persistence to fluoroquinolones is governed by more than the presence of TA systems and is not only a passive-by product of arrested growth state (Bernier *et al*. 2013), as described above (Goormaghtigh and Van Melderen 2019). It is plausible to assume that both types of growth arrested and growth-independent (specialist) fluoroquinolone persisters could co-exist in an isogenic culture and that their ratio would fluctuate depending on environmental/culture conditions.

### Transporter-linked persistence

Increasing intracellular antibiotic uptake has been shown to lead to elimination of persister cells is some systems (Allison, Brynildsen and Collins 2011). Subsequently, it was demonstrated that, when treated with β-lactam antibiotics, *E. coli* persister cells show higher efflux pump activity compared to non-persister cells and accumulate lower levels of drug intracellularly (Pu *et al*. 2016). Heterogeneity of uptake mechanisms and efflux pumps may thereby be another source of persisters. Several time-kill studies have also confirmed that increasing efflux pump activity can decrease the number of persisters (de Steenwinkel *et al*. 2010; Caleffi-Ferracioli *et al*. 2016; Pu *et al*. 2016).

### Intrinsic asymmetry of cell division and persistence

Bacteria reproduce asexually through binary fission, during which a ‘mother’ cell first duplicates its genetic material, then moves a copy of the bacterial chromosome to each cell pole and afterwards splits in half into two ‘daughter’ cells by synthesis of new cell wall. This mechanism of cell division results in an intrinsic asymmetry since each daughter cell inherits an ‘old pole’ (from the mother cell) and a ‘new pole’ (synthesized during division). Further fission events will then result in some cells accumulating ‘old poles’ (‘old pole daughters’ of ‘old pole mothers’) (Lapinska *et al*. 2019). Research on *E. coli* has shown that these old pole cells tend to have lower growth & glucose accumulation rates when compared to their ‘new pole’ sisters and that this effect is cumulative until the ‘old pole’ lineage reaches an apparent asymptote (in other words the change accumulation with subsequent generations reaches near-zero). The underlying mechanism behind this phenomenon is currently unknown, but does not appear to simply be caused by an accumulation of old & misfolded protein aggregates at the ‘old pole’, as previously hypothesized (Rang, Peng and Chao 2011; Lapinska *et al*. 2019). Whatever its origin, since asymmetric cell division appears to be a source of asymmetry in growth rate, it is potentially a source of persistence.

**Figure 6.**
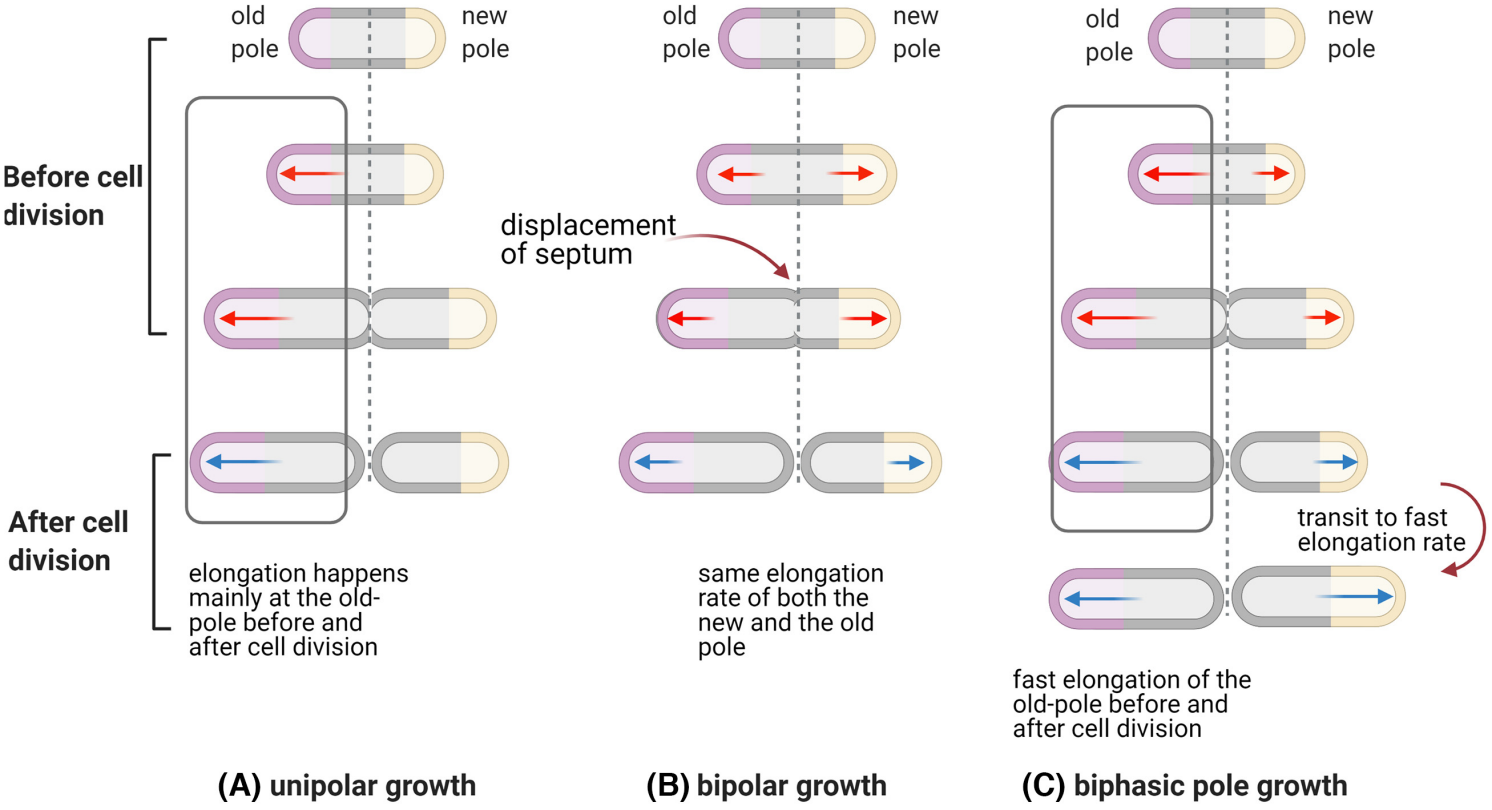
Elongation and division of M. smegmatis produces asymmetric bacterial cells (created with Biorender.com) **(A)** In the unipolar growth model, cells with the new pole are smaller and grow slower than those that inherited the old pole (Aldridge *et al*. 2012). **(B)** In the bipolar growth model, the old pole daughter has a similar growth rate prior to and after division, but is a larger size due to displacement of the septum at cell division (Santi *et al*. 2013; Wakamoto *et al*. 2013). **(C)** In the biphasic growth model, the new pole inheritor grows slower than the old pole daughter and transits to fast-growth before the next cell division due to variable duration of lag phase (Hannebelle *et al*.^2020^ ). Red arrow: growth rate of cell pole at cell division, Blue arrow: growth rate of cell pole after cell division.

Mycobacteria appear to undergo asymmetric cell division to an even greater extent than *E. coli*. In a pioneering single cell imaging study, *M. smegmatis* cells were demonstrated to grow faster and bigger in the old-pole inheritor (i.e. from the ‘grandmother’) and slower and smaller in the new-pole daughter. However, susceptibility appeared to be antibiotic-specific, with shorter, slower cells exhibiting increased tolerance to cell wall synthesis inhibitors such as cycloserine or meropenem and old pole larger, faster cells exhibiting enhanced tolerance to the RNA-polymerase inhibitor rifampicin (Aldridge *et al*. 2012). This heterogeneity was determined to be dependent upon key proteins, such as the scaffold protein Wag31 and loss of asymmetry protein Lam (Rego, Audette and Rubin 2017), the role of which, along with others, have been expertly reviewed recently (Longsdale and Aldridge 2018). Asymmetric distribution during cell division of irreversibly oxidized proteins (IOPs) in both *M. smegmatis* and *M. tuberculosis* also resulted in heterogeneity of growth rate, with daughter cells inheriting more IOPs growing more slowly and showing increased susceptibility to antibiotics (Vijay *et al.*^2017^). Moreover, as mentioned above, susceptibility to the pro-drug isoniazid was determined to be independent of the growth rate of single cells and instead, related to stochastic expression of the *KatG* gene, which encodes an isoniazid activating catalase peroxidase (Wakamoto *et al*. 2013). Alongside antibiotic-specific responses, the growth medium or conditions (i.e carbon source) may also affect asymmetry in a manner dependent upon pole age (Priestman *et al*. 2017). Other studies found that growth rate is independent of pole age and suggest that cell-size at birth (L_0_) is key with both cells elongating at the same rate post division (Santi *et al*. 2013; Wakamoto *et al*. 2013). These models are diagrammatically represented in Fig. 6 and include a recent biphasic pole growth model which centres upon the variable lag phase providing growth heterogeneity (Hannebelle *et al*. 2020). Regardless of the model, it is clear that a considerable degree of cell asymmetry, and hence growth rate and size, is characteristic of mycobacteria and may provide a reservoir for the phenotypic diversity that generates persistence. Moreover, the clinical relevance of heterogeneity has also been demonstrated in bacilli grown from TB patient sputum samples with larger, faster cells exhibiting increased tolerance to rifampicin and oxidative stress (Vijay, Vinh and Hai, 2017). It appears that in mycobacteria, persisters may be antibiotic specific and the high heterogeneity resulting from asymmetry may be one of the reasons for the high antibiotic persistence noted in mycobacterial infections.

## ORIGIN OF PHENOTYPIC HETEROGENEITY OF BACTERIAL POPULATIONS AS A SOURCE OF ANTIBIOTIC PERSISTENCE

### Noise in gene expression levels leads to phenotypic heterogeneity

It has been apparent since the advent of single cell studies that clonal populations of cells exhibit stochastic variation, also known as noise, in the expression of genes (Elowitz *et al*. 2002; Cai, Friedman and Xie 2006; Levine and Hwa 2007). The causes of this variation are largely unknown, but the amount of noise in any system is inversely proportional to the copy number of molecules controlling the system, so very low copy numbers of many cellular components such as regulatory molecules and structural molecules (Guptasarma, P. 1995) will lead to high noise levels in phenotypes controlled by low copy number molecules. Random partitioning of low copy number molecules at cell division is also a source of phenotypic variation (Huh and Paulsson 2011). Moreover, ergodicity-breaking, essentially incomplete mixing of the cell so that it does not sample all its possible microstates within one division cycle, has also been proposed to be a factor responsible for phenotypic heterogeneity (Rocco, Kierzek and McFadden 2013).

Heterogeneity may be present in any cell phenotype but, given the strong association between growth rate and persistence, phenotypic variation that influences growth rate is highly likely to influence the frequency of persisters in a population. That growth rate heterogeneity exists is apparent from many studies and is illustrated in Fig. 2 which shows the distribution of growth rate in a population of *E. coli* cells taken from a microfluidics experiment performed in our own laboratory.

Growth rate not only varies widely within the population but also, and rather strikingly, along cell lineages, including the persister family lineages as can be seen in Fig. 2, which illustrates growth rate of single *E. coli* cell lineages tracked across multiple generations. As can be seen, the growth rate of daughter cells varies markedly from growth rate of their mother cell. In fact, the correlation of growth rate between mother and daughter cells is very close to zero (Hingley-Wilson *et al*. 2020). This is remarkable since each pair of daughter cells is derived, by binary fission, from a single mother cell, so would be expected to epigenetically inherit cellular constituents controlling the mother's growth rate, such as copy number of key enzymes, number of ribosomes etc. However, whatever the cause, cell division is clearly an engine for the continuous generation of cells with widely varying growth rates, including very slow growing cells that might be spontaneous or triggered persisters (i.e. growth rate dependent).

The source of epigenetic variation in growth rate is currently unknown, but it is well established that a myriad of genes can influence growth rate, as is apparent from the commonplace observation that mutation or induced changes in gene expression very often lead to altered growth rates. It thereby seems likely that stochastic variation in expression of many genes will cause stochastic variation of growth rate. Note also that, in the mutational screen that led to the isolation of the original *E. coli* hip mutants including the hipA7 strain (Moyed and Bertrand 1983), all of the putative hip mutants grew slower than the wild-type in the rich media. As already described, the phenotype of the intensively studied hipA7 mutant is caused by a mutation in a toxin-antitoxin system whose expression depresses growth rate.

The hypothesis that persistence is due to epigenetic variation in growth rate is supported by recent results from our own laboratory, which has identified control of epigenetic inheritance as a potential source of persisters (Hingley-Wilson *et al*. 2020). The research studied the hipQ mutant of *E. coli*, isolated in during mutagenesis screen in 1990, which displays increased spontaneous persisters when exposed to norfloxacin and ampicillin (Wolfson *et al*. 1990). Single cell growth studies across several generations established that the high persister phenotype of hipQ is associated with the novel phenotype of *reduced phenotypic inheritance* (RPI), identified as reduced correlation of growth parameters such as division time, size at birth or cell elongation rate, either between mothers and daughter cells, or between sister cells. The results suggested that genes influencing epigenetic inheritance play a role in persister cell formation. The study also identified the locus of the hipQ phenotype as a mutation in a gene, *ydcI*, that encodes a putative transcription factor (Hingley-Wilson *et al*. 2020).

### A hunker theory of persistence

Aforementioned studies tend to argue against a single mechanism of persistence prompting a more general theory of persistence. However, growing slowly, or not at all ("dormancy") has been shown to induce tolerance, particularly to cytotoxic antibiotics. For example, Toumanen and colleagues determined that the rate of killing of *E. coli* cultures exposed to β-lactams was directly proportional to their growth rate, with slower growing cultures exhibiting enhanced tolerance (Tuomanen, Cozens and Tosch 1986).

For antibiotics to reach their target, they need to penetrate the cell and bind to their target. Cells that, for a variety of reasons, *hunker down* by growing more slowly, metabolising more slowly, transporting materials across their cell membranes more slowly or activating antibiotics more slowly, will be killed more slowly leading to a state of persistence.

This hunker theory of persistence is consistent with the finding that persisters are not necessarily slow growing, as in the specialised class of persisters (Fig. 4). Clearly, not all slow-growing cells are persisters; and not all persisters are slow growing. Slow growth may predispose towards the state of persistence; but it is neither sufficient nor necessary. Just as there are many possible ways of hunkering down to survive a storm (sheltering in the basement, shutting the windows, reinforcing the roof), there may be many ways to survive the onslaught of antibiotics. Indeed, it seems likely that any stochastically-varying factor that varies growth, metabolism, drug penetration or activity in individual cells, is likely to be capable to initiating a persister state and that these may be antibiotic specific (i.e. the SOS response after removal of ofloxacin) (Goormaghtigh and Van Melderen 2019).

## IS IT POSSIBLE TO DEVISE THERAPIES THAT TARGET THE PERSISTENCE STATE?

### Clinical application of persister-targeting treatment regimes

Until recently, little attention had been given specifically to the elimination of persisters in the clinical setting, however some antibiotics which were designed to treat drug resistance have also been found to shorten treatment duration, suggesting that the elimination of persisters in clinical infections can play a role in enhancing treatment efficacy. Two new licensed drugs for TB treatment - bedaquiline (BDQ) and delamanid (DLM) which have been used to treat multidrug-resistant *M. tuberculosis* since 2012 and 2014 (Zumla *et al*. 2014), also showed potential in eliminating persistence in clinical treatment. BDQ binds to the a and c subunits of the F_0_ domain of the ATP synthetase, thus inhibiting ATP synthesis and causing cell death in both replicating and metabolically-active non-growing mycobacteria (Goulooze, Cohen and Rissmann 2015). A BDQ-modified regimen could achieve total organ CFU count clearance in the *M. tuberculosis*-infected mice after 8 weeks, much faster than the standard regimen (rifampicin, isoniazid, pyrazinamide and ethambutol) which takes 14 weeks on average (Hu *et al*. 2019). DLM inhibits the synthesis of mycolic acids, which disrupts the formation of cell wall and facilitates drug penetration into mycobacterial cells (Liu *et al*. 2018). DLM also showed bactericidal activity against non-growing cells (although at higher concentrations) and shortened treatment duration of chronic TB disease in the guinea pig model (Chen *et al*. 2017).

Since discovery of entirely new drugs is notoriously difficult, repurposing antibiotics has been proposed as an alternative strategy to identify effective drugs. Some regimens which are used to treat other diseases have also been shown to be effective in the killing of persisters. Mitomycin C, a former cancer drug, was shown to be effective on both actively growing cells and non-growing cells through spontaneous cross-linking of DNA in bacteria. Mitomycin C is more effective than ciprofloxacin and ampicillin in killing persisters of enterohaemorrhagic *E. coli, S. aureus* and *P. aeruginosa* within laboratory culture as well as in the Lubbock chronic wound pathogenic biofilm model, a model which closely represents growth conditions of polymicrobial infections (Kwan, Chowdhury, and Wood 2015). Therefore, mitomycin C could potentially be a broad-spectrum compound used to eliminate persisters in the treatment of recalcitrant infections (Kwan *et al*. 2015). Unfortunately, like many cancer drugs, mitomycin C treatment can cause multiple side-effects, notably hair loss, nausea and vomiting, leukopenia & thrombocytopenia or in some cases pneumonitis and haemolytic-ureaemic syndrome (NICE 2020) and therefore development of less ‘invasive’ persister treatment regimens is still urgently needed.

**Figure 7.**
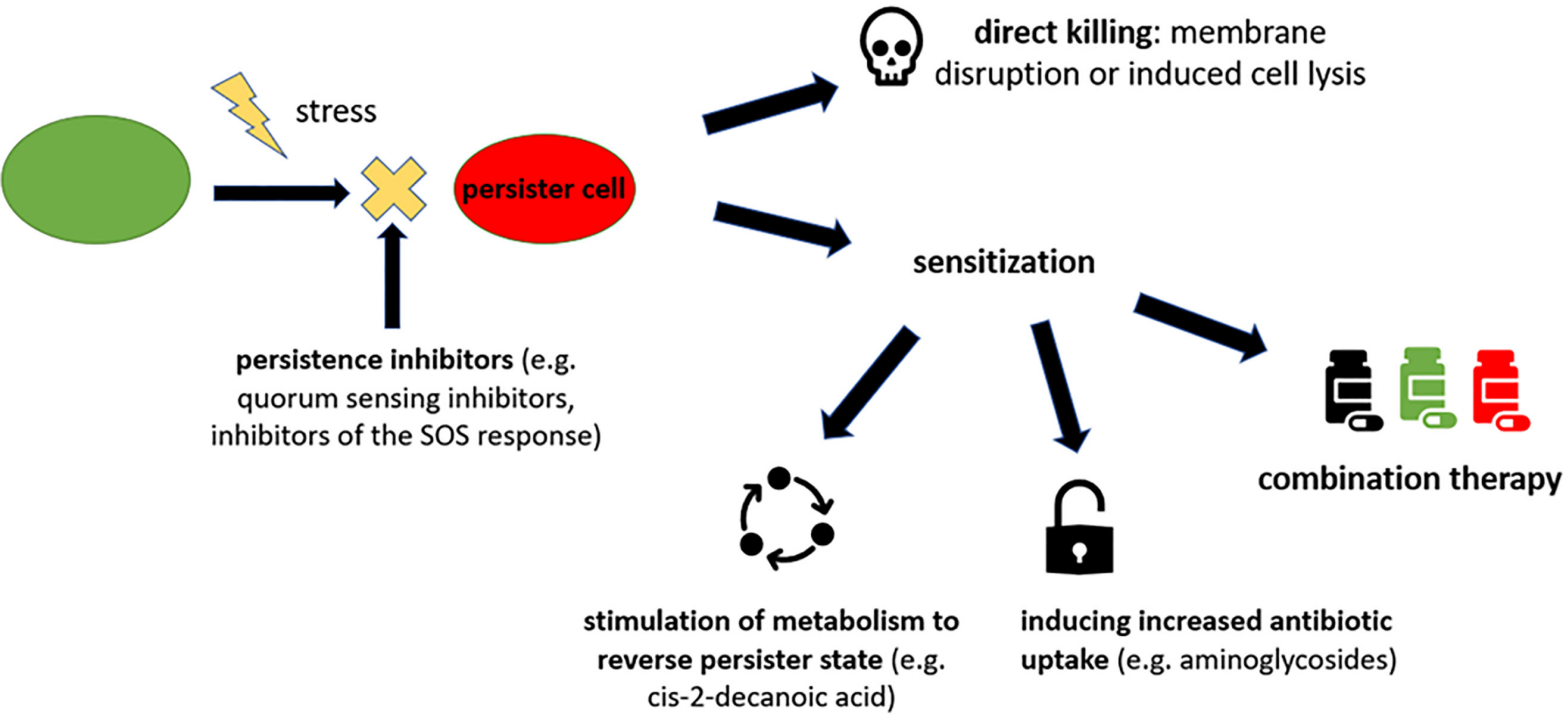
Overview of current strategies tackling the presence of persister cells. Several strategies are displayed here which have been developed that target persisters specifically through direct killing, sensitization to antibiotics or by prevention of persister cell formation.

Although only a few compounds specifically targeting persisters have been used in a clinical setting, several potential strategies of persister cell elimination have been proposed, including direct killing of persisters, sensitization of persisters to antibiotics and inhibition of persister cells formation (Fig. 7). The noted high persister hipAB clinical strain (Schumacher *et al*. 2015) and the link between persisters and the development of genetic AMR makes targeting persisters even more of a priority. Conversely, research into mechanisms and gene mutations responsible for generating low frequency persister strains or cultures may lead to identifying drug targets that reduce persister formation, yet is an area that is often overlooked.

### Direct killing of persister cells can be achieved by membrane disruption or induced lysis

Several compounds have been used *in vitro* to kill persisters directly. For example, a quinolone-derived compound HT61 has been found to have a synergistic effect when used with neomycin, gentamycin and chlorhexidine (antiseptic agent) in both MSSA and MRSA treatment (Hu and Coates 2013). HT61 depolarises bacterial cell membrane through interaction with the lipid bilayer, resulting in leakage of cytoplasmic proteins and ATP from the cell, thus being effective against both actively growing (‘specialised’) and & growth-arrested persister cells (spontaneous and triggered) (Hubbard *et al*. 2017). Brilacidin, a kind of host defence protein mimetic (a synthetic peptide which mimics natural hosts defence peptides), which is under phase II clinical trial for the treatment of serious skin infection, causes membrane depolarization in both Gram-positive and Gram-negative bacteria, like *S. aureus* (Mensa *et al*. 2014) or *E. coli* (Mensa *et al*. 2011). It also shows bactericidal activity against non-replicating bacteria (Mensa *et al*. 2014). Phage-derived lysins, such as endolysin LysH5 produced by a *Staphylococcal* bacteriophage phi-SauS-IPLA88, are another source of compounds capable of killing both replicating and non-replicating bacteria (Garcia *et al*. 2010). Treatment with this endolysin was able to eliminate planktonic *Staphylococcus* persisters *in vitro* remaining after treatment with ciprofloxacin and rifampicin so it is a potential adjuvant candidate for those antibiotics. However, more research is required *in vivo* to confirm the potential of these effector molecules (Gutierrez *et al*. 2014). Another candidate molecule, polycationic glycopolymer (PAAG) induces rapid permeabilization of the cell membrane, causing membrane depolarization and cell death of *P. aeruginosa* persisters, as well as in actively-growing cells (Narayanaswamy *et al*. 2018).

**Table 1.** Terms used commonly in persistence research.

**antibiotic persistence**	A phenotypic feature defining a subpopulation of isogenic bacterial cells which display a greatly reduced killing rate to antibiotic(s) when compared to the entire population; the reduction of the killing rate is largely independent of the antibiotic concentration (i.e. no change in minimum inhibitory concentration (MIC) to the antibiotic is observed); also referred to as **subpopulation tolerance** and **heterotolerance** (Balaban *et al*. 2019)
**antibiotic tolerance**	A feature defining an entire population of bacterial cells which display decreased killing rate to antibiotic(s); similarly to persistence the reduction of killing rate is largely independent of the antibiotic concentration (Balaban *et al*. 2019)
**antibiotic/antimicrobial resistance (AMR)**	A feature of the entire population of microorganisms which possess a genetic resistance mechanism that allows them to overcome the harmful effect of a given antibiotic; AMR is concentration dependent and characterised by an increase in MIC for a given antibiotic (Cantón and Morosini 2011)
**persistent (chronic) infection**	Any infection (bacterial/viral/fungal) which persists in the host for a prolonged period (Centre for Disease Control and Prevention 2019)

### Persistence can be reversed by ‘kick-starting’ cellular metabolism or increasing antibiotic uptake

Persisters with reduced growth rate and downregulated metabolism significantly increase the time required for many antibiotics to achieve a desired killing effect. Induction of metabolism or growth of persisters can thereby facilitate the action of antibiotics. A fatty acid signaling molecule- cis-2-decenoic (cis-DA) can increase overall metabolic activity of *P. aeruginosa* persisters (measured as respiratory activity) (Marques *et al*. 2014). Combination of cis-DA with ciprofloxacin resulted in up to a 2-log culturable cell number reduction when compared to treatment with ciprofloxacin alone (Marques *et al*. 2014).

Alternatively, promotion of antibiotic uptake will increase its intracellular concentration particularly in persisters that display increased efflux activity. Efflux pump inhibitors (EPIs) could effectively eliminate efflux-dependent persisters (Kvist, Hancock and Klemm 2008). Although no EPIs have been put in clinical practice, a great number of EPIs like Carbonyl Cyanide-m-Chlorophenylhydrazone (CCCP) or phenylalanyl arginyl β-naphthylamide (PAβN) showed synergistic effect with antibiotics *in vitro* (Singh *et al*. 2011; Lamers, Cavallari and Burrows 2013). Hypoionic shock could also facilitate uptake of aminoglycosides by increasing proton motive force (PMF) of both nutrient shift and nutrient starvation-induced *E. coli* persister cells (Chen *et al*. 2019). Although hypoionic shock is not feasible in the clinic, developing alternative PMF drug-uptake inducing strategies could be effective against persisters. Indeed, aminoglycoside uptake through PMF has also been demonstrated in persister cells though metabolic stimuli such as mannitol and fructose in *E. coli, P. aeruginosa* and *S. aureus* (Allison, Brynildsen and Collins 2011; Barraud *et al*. 2013). Although most of these compounds are currently only researched in a laboratory setting and might not be practical in a clinical practice, exploration of novel compounds could be the stepping stone for development of new effective regimens.

### Is combination therapy the ‘holy grail’ of persistence treatment strategies?

Combination therapy is an effective approach that has been used to target persisters as it would enable differential killing of naturally heterogenous persisters and potentially inhibit the generation of genetic AMR from persister cells. For instance, treating *Borellia burgdoferi* (the causative agent of Lyme disease) with a combination of doxycycline (bacteriostatic, active against growing cells), cefoperazone (β-lactam) and daptomycin (which disrupts membrane potential and is active against both growing and dormant cells) was effective in eradicating persister cells which had previously been able to survive the standard doxycycline treatment. Combinations of these three antibiotic classes *in vitro* was more effective against persisters than any of the compounds alone or in dual combinations (Feng *et al*. 2015). Similarly, combining erythromycin, which is known to inhibit quorum sensing pathways and motility in *P. aeruginosa*, with colistin (a membrane active antibiotic) eliminated persister cells in *P. aeruginosa* biofilms (Chua *et al*. 2016) (Baek *et al*. 2020).

Notably, active *M. tuberculosis* infection is routinely treated with a combination of isoniazid, rifampicin, pyrazinamide and ethambutol in order to eradicate infection (WHO 2019). Pyrazinamide (PZA) was found to shorten the treatment of TB from 9–12 months down to 6 based on clinical trials and animal studies, indicating that it is likely targeting persister cells. The target of PZA has recently been identified (Sun *et al*. 2020) as PanD, which is an aspartate decarboxylase involved in the pantothenate biosynthetic (Sun *et al*. 2020). However, the exact mechanism and whether PZA targeting of PanD reduces mycobacterial persisters remains to be determined.

### Inhibition of persister cell formation

Inhibition of signalling pathways involved in the establishment of the persistence state under stressful environmental conditions is another potential strategy of targeting persistence. However this is a challenging task as persisters can form through numerous often redundant routes. Nevertheless, this approach has shown some promising results. For example, Vitamin C (which is not bactericidal) at high concentrations showed an inhibitory effect on the synthesis of (p)ppGpp *in M. smegmatis*, which the authors hypothesised could potentially stall long-term infection and persistence in *M. tuberculosis* (Syal, Bhardwaj and Chatterji 2017). Interestingly, a recent seminal study on Vitamin C has determined its capacity to reduce treatment time with front-line drugs including isoniazid or rifampicin *in vivo* in a murine model of tuberculosis (Vilcheze, Kim and Jacobs 2018). This study determined that enhancing *M. tuberculosis* respiration via the addition of Vitamin C or N-acteylcysteine prevented persister formation (Vilcheze *et al*. 2017). Another compound, relacin, a RelA inhibitor capable of reducing (p)ppGpp synthesis, decreased cell viability of *Streptococcus pyogenes in vitro* (Wexselblatt *et al*. 2013). Although more supporting evidence of *in vivo* efficacy of RelA inhibitors is needed, a recent study utilising a dental root infection model has shown that relacin combined with NaOCl (commonly used in dental practice as disinfectant) was effective at eradicating *Enterococcus faecalis* biofilms without displaying cytotoxicity to human cells (Cai *et al*. 2018). The study provides a proof of concept of the principle that compounds which inhibit persister cell formation can be effective components of treatment strategies.

## SUMMARY

The phenomenon of antibiotic persistence is intimately linked with phenotypic heterogeneity, which may be critical for survival of microbial populations. Persistence has been linked to treatment failure since its discovery over 70 years ago, and more recently has been suggested to be a ‘stepping-stone’ for the emergence of genetic resistance (Windels *et al*. 2019; Liu *et al*. 2020). Heterogeneity is also rife in the low frequency persister subclass, which includes spontaneous and triggered growth-dependent and specialised growth-independent phenotypic variants. However, much remains unclear, such as how different mechanisms involved in the establishment of the persister state are regulated (if at all). Persister cells appear to be generated by a great variety of mechanisms as described here, leading to the hunker theory of persistence in which the phenomenon is associated with stochastic variation of factors influencing growth, drug penetration, metabolism or the SOS and stringent response. Since the acknowledgment of the clinical importance of persisters, multiple treatment strategies have been developed to target persister cells, inhibit their formation or ‘wake them up’ from the persister state; however, the efficacy of these strategies *in vivo* requires validation. This highly heterogenous and clinically relevant subclass may require simultaneous approaches to target each class of persister in order to subvert persistence and hinder the development of genetic resistance.
